# Molecular Characterization of Ca^2+^/Calmodulin-Dependent Protein Kinase II Isoforms in Three Rice Planthoppers—*Nilaparvata lugens*, *Laodelphax striatellus*, and *Sogatella furcifera*

**DOI:** 10.3390/ijms20123014

**Published:** 2019-06-20

**Authors:** Wei-Xia Wang, Feng-Xiang Lai, Pin-Jun Wan, Qiang Fu, Ting-Heng Zhu

**Affiliations:** 1 State Key Laboratory of Rice Biology, China National Rice Research Institute, Hangzhou 310006, China; wangweixia@caas.cn (W.-X.W.); laifengxiang@caas.cn (F.-X.L.); wanpinjun@caas.cn (P.-J.W.); 2 College of Biological and Environmental Engineering, Zhejiang University of Technology, Chaowang Road, Hangzhou 310014, China

**Keywords:** *Nilaparvata lugens*, *Laodelphax striatellus*, *Sogatella furcifera*, Calcium/calmodulin-dependent protein kinase II, RNAi

## Abstract

This study reports the identification of splice variants for the calcium/calmodulin-dependent protein kinase II (*CaMKII*) gene from *Nilaparvata lugens*, *Laodelphax striatellus*, and *Sogatella furcifera*. CaMKII is a multifunctional serine/threonine protein kinase that transduces Ca^2+^ signals in cells to control a range of cellular processes in the nervous system and muscular tissue. Sequence analysis showed that CaMKII was 99.0% identical at the amino acid level among three rice planthoppers, with the exception of a variable region located in the association domain. Four kinds of 20–81 amino acid “inserts” were found in the variable region. The phylogenetic tree of the deduced amino acid sequences showed that the NlCaMKII isoforms were more closely related to the LsCaMKII isoforms and were slightly distinct from SfCaMKII. CaMKII-E was the dominant type among the five main isoforms. *CaMKII* genes were constitutively expressed in various nymphal and adult stages and in tested tissues with the predominant transcription occurring in the head. There was no major tissue specificity of isoform expression, but the expression pattern and relative abundance of isoforms varied when compared with the RT-PCR between tissues. In addition, RNAi in *N. lugens* with dsRNA at a concentration of 200 ng nymph^−1^ induced a mortality of 77.7% on the 10th day and a reduction in the mRNA expression level of 67.2%. Unlike the holometabolous insect *Helicoverpa armigera*, the knockdown of *NlCaMKII* did not suppress the expression of 20E response genes, such as *ECR*, *USP1*, and *HR3*, in *N. lugens*. These results indicate that the role of CaMKII in hemimetabolous insects may be different from that in holometabolous insects.

## 1. Introduction

Rice (*Oryza sativa* L.) is a major food crop in Asia. However, it also serves as an important food source for insects and is attacked by around 800 species [1]. Of these insects, the brown planthopper (*Nilaparvata lugens* (Stål)), the white-backed planthopper (*Sogatella furcifera* (Horváth)), and the small brown planthopper (*Laodelphax striatellus* (Fallén)) are three major destructive pests in rice ecosystems. They belong to Hemiptera: Delphacidae and go through five nymphal stages to become adults. Among them, *N. lugens* is a monophagous pest that feeds only on rice plants, whereas *S. furcifera* and *L. striatellus* are oligophagous pests, feeding mainly on rice plants but also on many other Gramineae plants [2]. These species damage rice plants through inserting their styles into the vascular tissue of leaf blades and leaf sheaths and ingesting the sap, causing a colossal loss in yield, the transmittance of virus diseases, and also making the plants vulnerable to bacterial and fungal attacks. Heavy infestation can cause the complete drying and wilting of plants, known as “hopperburn” [3]. Due to the extensive but unplanned applications of insecticides, almost all strains of these pests have become resistant through adaptive mutation [4,5,6]. Therefore, in order to develop alternative and novel strategies to control insect pests that are as equally effective as conventional insecticides without affecting the environment, it is necessary to explore and understand the molecular mechanisms of pest biology and the dynamic interactions between plant and pest.

Ca^2+^/calmodulin-dependent protein kinase II (CaMKII) is an oligomeric Ser/Thr protein kinase that is activated by calcium-bound calmodulin [7]. CaMKII mediates the Ca^2+^ signaling by phosphorylating a wide variety of substrate proteins involved in multiple important physiological and pathological processes [8]. In *Blattella germanica*, the expression of CaMKII mRNA in DDT (dichloro-diphenyl-trichloro-ethane) or pyrethroid-resistant strains was lower than that in susceptible ones, this reduction indicates that CaMKII may play important roles in DDT or pyrethroid resistance [9]. In *Drosophila*, it was demonstrated that increasing the level of constitutively active CaMKII decreased the initial courtship and enhanced the rate of the suppression of courtship in response to a mated female. This effect could be mediated by cholinergic neurons that enhance training-dependent suppression [10,11]. In *Bombyx mori*, it was found that a species-specific sex pheromone released by female moths to attract conspecific male moths is regulated by a neurohormone. The signal pathway of the neurohormone involves phosphorylation by CaMKII [12]. In *Helicoverpa armigera*, CaMKII showed an increased expression and phosphorylation during metamorphosis. *CaMKII* knockdown by dsCaMKII injection into the larvae prevented the occurrence of larval–pupal transition and suppressed the 20-hydroxyecdysone (20E) response gene expression [13]. The importance of CaMKII in holometabolous insects and neuronal processes is well documented [10,11,14,15,16].

There exists a diversity of CaMKII isoforms in mammals, four *CaMKII* genes in humans, termed α, β, γ, and δ, give rise to 40 isoforms through alternative splicing [17]. For *Drosophila* [18] and *Periplaneta americana* [19], up to nine and five closely related isoforms were identified, respectively, which are encoded by the alternative splicing of a single gene. CaMKII in *Drosophila* is 77% identical to the rat α subunit, which is distributed only in neurons and expressed at high levels in forebrain neurons and is known to play important roles in the developmental and physiological modulation of the nervous system [20]. To date, little is known about the number of CaMKII isoforms, tissue distribution, and their potential function in hemimetabolous insects. Thus, in the present study, we cloned, sequenced, and characterized the tissue-distribution pattern of CaMKII isoforms in three rice planthopper species. Additionally, the RNAi efficiency of *NlCaMKII* by injecting dsRNA into the insect body and the nymph lethality of the RNAi in *N. lugens* were analyzed.

## 2. Results

### 2.1. Identification of CaMKII Isoforms and Sequence Analysis

**Figure 1:**
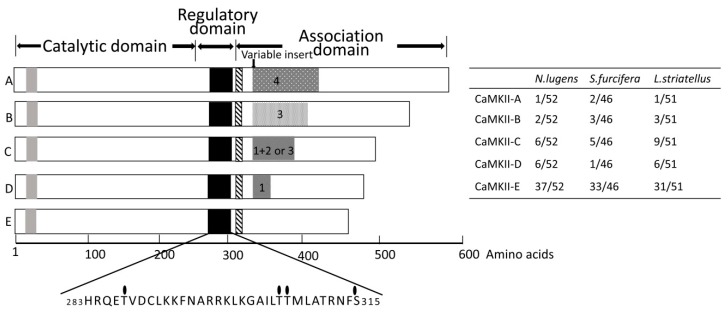
Schematic representation of five main isoforms of the CaMKII polypeptide in three rice planthoppers. The gray-filled box is the putative ATP-binding site. The different dotted boxes show the insertion of amino acid sequences named 1, 2, 3, and 4. The thick shadowed box shows two different (KSG or SKYDVQG) motifs at the beginning of the association domain. The expanded view of the domain structure of the CaMKII sequence indicates the important features of the regulatory domain. The filled ovals show the autophosphorylation sites within the regulatory region that prevent the re-binding of Ca^2+^/CaM. The numbers of clones of each isoform with respect to the total number of isolated clones are given in the table on the right.

Based on the transcriptome data of three planthopper species, PCR primers were designed and used to clone the *CaMKII* gene from RNA isolated from three planthopper species. To obtain alternative transcripts, about 50 positive clones were sequenced in both strands from *N. lugens*, *S. furcifera*, and *L. striatellus*, respectively. Eleven, thirteen, and nine forms of cDNA coding for CaMKII were cloned and sequenced from *N. lugens*, *S. furcifera*, and *L. striatellus*, respectively. The open reading frame (ORF) of the cDNAs ranged from 1458 to 1719 bp in length. The translated peptide sequences of these CaMKII were used for Blastp searches of a local protein database containing NCBI non-redundant proteins with the defaulted algorithm parameters. The results revealed their strong similarities with other insect species and three domains (catalytic, regulatory, and association) were identified (Figure 1). CaM-binding and adjacent autoinhibitory domains (residue 285–315), as well as autophosphorylation site overlaps, lie at the C-terminal of the regulatory domain. Two main essential autophosphorylation sites (Thr-287 and Thr-306/Thr-307) regulating the kinase activity, as well as one additional autophosphorylation site, Ser-315, were well conserved.

**Figure 2:**
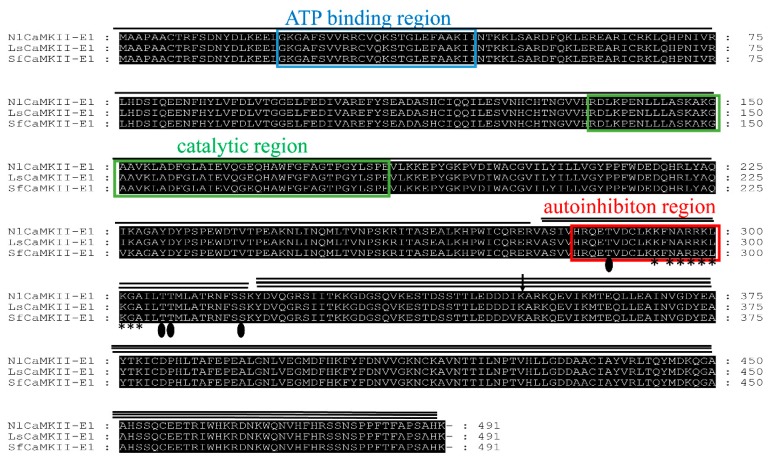
Amino acid sequence alignment of CaMKII-E1 proteins. The ClustalX (2.1) program was used for multiple sequence alignment. The catalytic, regulatory, and association domains are indicated by the single line, double line, and triple line, respectively. The blue and green boxes are the ATP-binding site and the catalytic region in the catalytic domain, respectively. The autoinhibition region in the regulatory domain is boxed in red. The asterisks and filled ovals show the CaM-binding region and the autophosphorylation sites within the regulatory region, respectively. The arrow represents the position at which various insert amino acids are inserted.

Sequence alignment indicated that the CaMKII with the shortest length was 99.0% identical at the amino acid level among the three rice planthoppers (Figure 2). Other sequences contain inserts located in the association domain that vary in size and the composition between the species. Four major inserts (hereafter referred to as Inserts 1, 2, 3, and 4) and the KSG or SKYDVQG motif were found at this site. The sequences were named CaMKII-A, B, C, D, and E according to the length and insert sequence. Within the same taxon, different members were marked with Arabic numerals to show the KSG or SKYDVQG motif at the beginning of the association domain. Among the five isoforms, CaMKII-E with no insert sequence was the most abundant form on the basis of the isolated clone number. The characteristics of CaMKII isoforms are listed in Table 1. The coding sequence (CDS) size and molecular weight of these kinase isoforms range from 486 to 572 aa and 54.8 to 64.0 kilodaltons, respectively. The sequences of the inserts in CaMKII from the three planthoppers are listed in Table 2. An amino acid sequence comparison between the CaMKII from the three rice planthoppers and the DmCaMKII isoforms showed a high level of homology ranging from 77.0% to 82.0%. Phylogenetic analysis with several CaMKII isoforms available in the database showed that the CaMKII isoforms from the rice planthoppers were distinct from *Drosophila*. NlCaMKII and LsCaMKII isoforms formed a cluster that was distinct from SfCaMKII. All isoforms from *S. furcifera* formed a small cluster (Figure S1). The details of GeneDB accession numbers were listed in Table S1.

**Figure 3:**
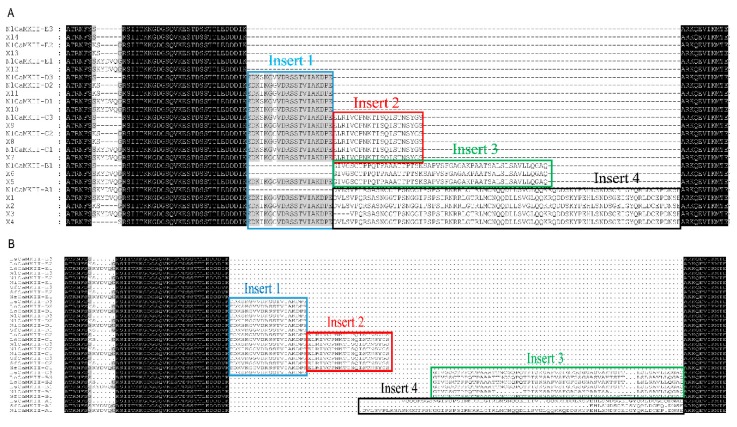
(**A**) Partial multiple alignment of CaMKII isoforms cloned by RT-PCR and fourteen predicted transcript variants (X1–X14) in *N. lugens*. (**B**) Partial multiple alignment of CaMKII isoforms cloned from *N. lugens*, *S. furcifera*, and *L. striatellus*. The different inserts are boxed with different colors.

The NlCaMKII isoform locates at scaffold 510 of the *N. lugens* genome and its coding sequences vary from 1458 to 1719 bp. Based on the *N. lugens* genomic sequence [21], up to 14 alternatively spliced products were predicted. Partial multiple alignment of eleven isoforms of NlCaMKII cloned by RT-PCR and the 14 predicted transcript variants (X1–X14) are shown in Figure 3A. At the protein level, the NlCaMKII-B, -C, -D, -E are identical to the transcript variants predicted by automated computational analysis. The transcript variant X5 containing Insert 1 and Insert 3 and transcript variants X1, X2, X3, and X4 containing Insert 1 and Insert 4 were not identified in our study.

### 2.2. Variable Insert Region of CaMKII in Rice Planthoppers

A similarity comparison was conducted between the insert sequences 1–4 to search for duplicated sequences. The result of this clustalW analysis is shown in Table S2. The similarity values above 60.0% are highlighted in bold. This comparison not only supports the groupings of similarly numbered variable domains between genes but also between different variable regions. Insert 1 in CaMKII from the three planthoppers is highly conserved. There are two different Insert 1s, in *N. lugens*, namely NlI1a and NlI1b, which have only two amino acids different from each other. NlI1a is the same as Insert 1 in *L. striatellus* and NlI1b is identical to that in the *N. lugens* genome predicted by automated computational analysis. Insert 1 codes for 20 amino acids and contains a CaM kinase consensus phosphorylation site, RSST. Insert 2 is identical among the three planthopper species. Insert 3 in *N. lugens* has 70.0% and 72.0% similarity with that in *S. furcifera* and *L. striatellus*, respectively. Insert 4 in CaMKII from the three planthoppers is different in each species. Insert 4 from *N. lugens* is encoded by two exons. One exon encoded sequence—EHLSNDSGEISYQRLDCEPDNSE—is identical with Insert 4 from *S. furcifera*. Partial multiple alignment of the CaMKII isoforms from *N. lugens*, *S. furcifera*, and *L. striatellus* is shown in Figure 3B.

### 2.3. NlCaMKII Gene Structure

**Figure 4:**
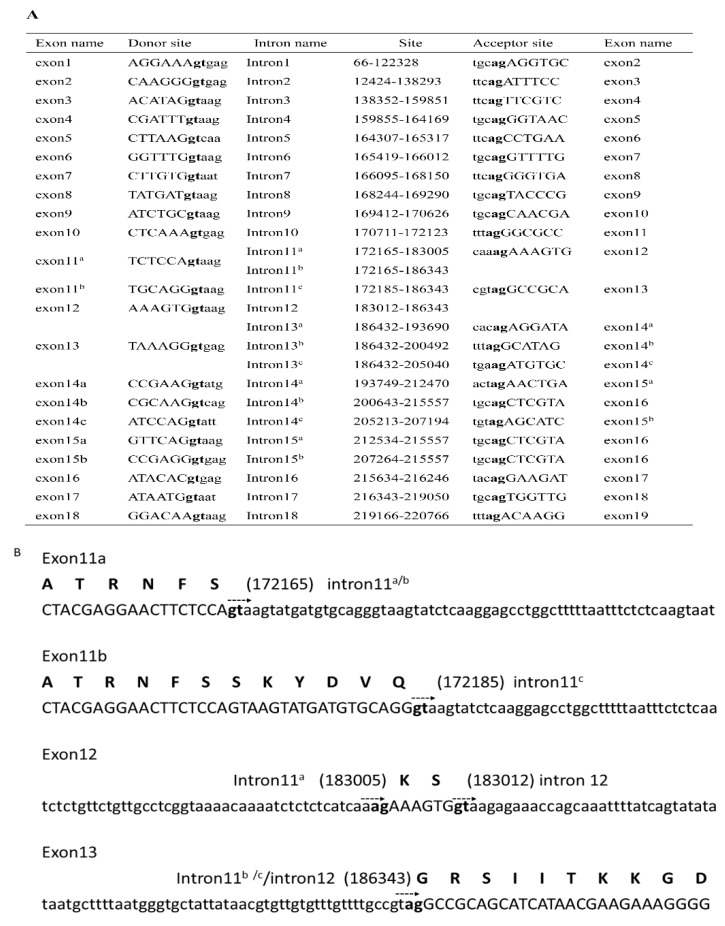
(**A**) Intron/exon organization of the *NlCaMKII* gene. (**B**) Differential splice sites in intron 11. Exon sequences are in capital letters, and intron sequences are shown in lowercase letters. Conserved gt and ag nucleotides are in bold lowercase letters. The arrows show the intron sequence direction from 5′ to 3′. The corresponding sites in the genome of the intron start or stop are shown in parentheses. The amino acid sequences are shown in bold uppercase letters.

To determine the structure of the *NlCaMKII* gene, we blasted the *N. lugens* genome database using a cloned cDNA sequence from *N. lugens*. Exon placement and total gene size were determined by comparing *NlCaMKII* cDNAs to genomic sequences. The *NlCaMKII* gene spanned approximately 220 kbp and contained 16–19 exons. The cDNA sequences of the identical regions of the eleven isoforms were composed of 10 highly conserved exons (exon 1 to exon 10) encoding the catalytic domain and 4 exons (exon 16 to exon 19) encoding the association domain. The variable domain was encoded by five independent alternate exons. Inserts 1, 2, and 3 were encoded by exon 14a, 15a, and 14b, respectively, while Insert 4 was encoded by two exons (14c and 15b). KS in the motif KSG was encoded by exon 12. SKYDVQ in the motif SKYDVQG was encoded by exon 11^b^. The intron and exon organization of *NlCaMKII* is listed in Figure 4A. Different 5′ and 3′ splice sites in intron 11 resulted in four types of exons—11a, 11b, 12, and 13 (Figure 4B).

### 2.4. Expression Pattern of CaMKII in Diverse Tissues and Stages

**Figure 5:**
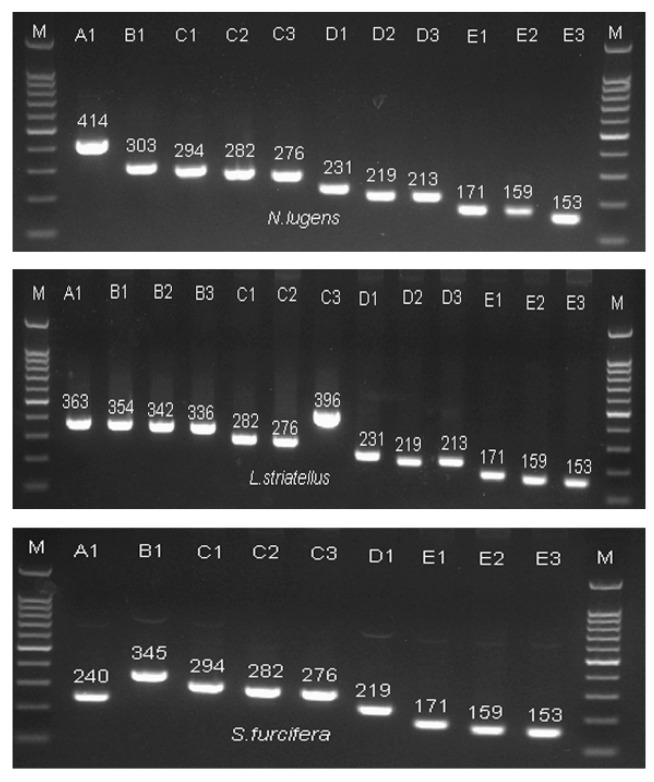
PCR products of the cloned cDNA sequences of *CaMKII* using 5′ and 3′ primers on either side of the nucleotide insertion site. M: DNA marker; A1–E3: the isoforms of *CaMKII* A1–E3; and Arabic numerals indicate the amplicon size.

To confirm the expression of each isoform, RT-PCR analysis was performed to amplify the fragment containing the variable region using primers flanking the variable region. A control experiment using cDNA clones as the template produced PCR products of various length, representing the mRNAs encoding the polypeptides of corresponding isoforms (Figure 5). This result strongly suggests that 11, 13, and 9 types of gene product are actually produced from *N. lugens*, *S. furcifera*, and *L. striatellus*, respectively. To test the amplify efficiency of RT-qPCR primer, cDNA diluted 1:5 fold along six serial dilutions was used to amplify the identical nucleotides in the catalytic domain of the three planthoppers. The efficiency of the qPCR primer for CaMKII is 98%, indicating the analytical method for quantitation was reliable.

**Figure 6:**
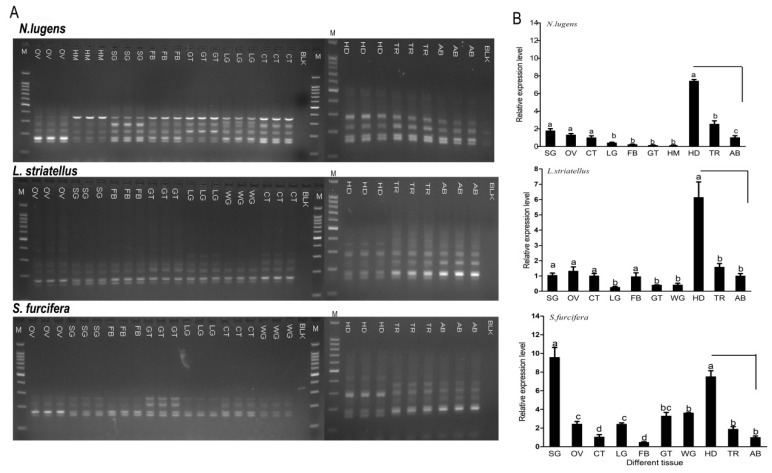
(**A**) RT-PCR products of cDNA templates prepared from total RNA prepared from different tissues and parts of *N. lugens*, *L. striatellus,* and *S. furcifera*. RT-PCR amplified with primer CaMKII-VR based on the common sequences around the variable region in *CaMKII*. (**B**) RT-qPCR analysis of cDNA templates prepared from total RNA prepared from different tissues and parts of *N. lugens*, *L. striatellus*, and *S. furcifera*. The RT-qPCR was analyzed with specific primer qCaMKII, which corresponded to identical nucleotides in the catalytic domain of *CaMKII*. The mRNA levels for each cDNA were normalized with respect to the 18s-rRNA mRNA level. Fold induction values of *CaMKII* genes were calculated with the ΔΔ*C*t equation and normalized to the mRNA level of *CaMKII* genes in the tissue cuticle or abdomen which were defined as 1.0. Each point represents the mean ± SE from three independent experiments. HM: hemolymph; FB: fatbody; SG: salivary glands; GT: midgut; OV: ovary; CT: cuticle; LG: leg; WG: wing; HD: head; TR: thorax; AB: abdomen and BLK: blank control with H_2_O as the template. As we could not obtain enough of a sample for the hemolymph extraction from either *L. striatellus* or *S. furcifera* and as the wings had been dissected from *N. lugens*, there are no expression data for these tissues.

The tissue expression analysis of RT-PCR showed that several bands were systematically observed in three parts of the body—the head, thorax, and abdomen and all tested tissues (Figure 6A). This means that the expression of *CaMKII* isoforms is multiple in all tissues, showing that there is no major tissue specificity of this isoform expression; however, the expression pattern and relative abundance of the isoforms varied among tissues. RT-qPCR analysis with specific primers corresponding to identical nucleotides in the catalytic domain of the three planthoppers showed that the transcript of *CaMKII* was more abundant in the heads than in the bodies. The expression amount in the tissues was different among the three planthopper species (Figure 6B). 

To test whether the *CaMKII* expression altered during nymph development, the stage expression analysis of *CaMKII* in the whole body of all instar nymphs and adult females and males of *S. furcifera* and *N. lugens* was detected via RT-PCR. The results showed that several isoforms were expressed in all the tested developmental stages (Figure S2A) and that the expression pattern of the isoforms was almost similar among the stages. The relative expressions of *CaMKII* were detected via RT-qPCR. The amount of *CaMKII* transcription slightly decreased after the 3rd and 2nd nymphs in *S. furcifera* and *N. lugens*, respectively (Figure S2B). 

### 2.5. Expression Profiling of NlCaMKII in Different Virulent Populations and Response to Resistant Rice 

To test whether the *NlCaMKII* expression pattern or amount altered among five *N. lugens* populations with different virulence levels in rice, the total RNA extracted from newly emerged adults was used as the template for the RT-PCR analysis. The results showed that several bands were systematically observed in all populations (Figure S3A) and that the expression pattern of the isoforms was similar among the populations. The RT-qPCR analysis showed that the amount of the transcript level of *NlCaMKII* was not significantly different among the five virulent populations (Figure S3B).

**Figure 7:**
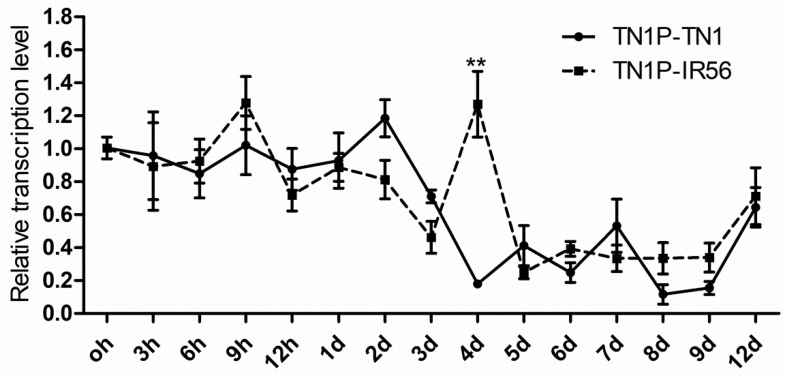
The transcription of *NlCaMKII* on the successive time of the TN1 population reared on two different rice varieties. Data are denoted as the mean ± standard error (SE) and analyzed by three-way ANOVA followed by Tukey’s honestly significant difference test. The mRNA levels for each cDNA were normalized with respect to the 18s-rRNA mRNA level. Fold induction values of *CaMKII* genes were calculated with the ΔΔ*C*t equation and normalized to the mRNA level of *CaMKII* genes in the newly emerged TN1 population, which were defined as 1.0. ** indicates the statistical difference by ANOVA followed by Duncan’s Multiple Comparison test (*p* < 0.01). TN1P-TN1 indicates that the TN1 population fed on the Taichung Native1 rice variety; TN1P-IR56 indicates the TN1 population fed on the resistant rice variety IR56.

In order to compare the *CaMKII* expression of *N. lugens* in susceptible and resistant rice varieties, the induced expression of the *NlCaMKII* in the TN1 population of *N. lugens*, which is incapable of breaking down the resistance of rice varieties containing resistance genes, was evaluated by rearing it on two contrasting rice varieties—IR56 (a *N. lugens*-resistant rice variety containing Bph3) and Taichung Native 1 (TN1, a *N. lugens*-susceptible rice variety). The effects of the rice varieties and the infestation periods on the transcription of NlCaMKII in the TN1 population was analyzed by RT-qPCR. A significantly induced expression in the TN1 population reared on IR56 rice on the 4th day was observed (Figure 7).

### 2.6. Gene-Silencing Effects of NlCaMKII on Development of N. lugens

**Figure 8:**
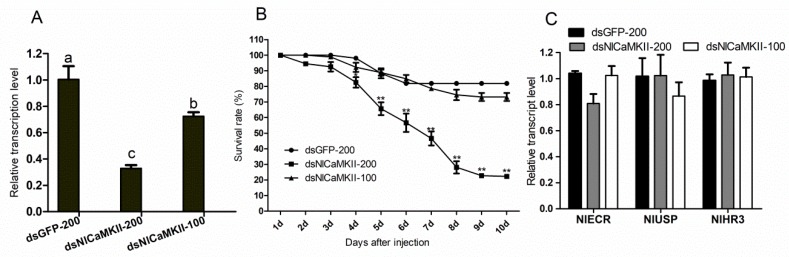
(**A**) The mRNA expression levels of *NlCaMKII* at the 4th day after injection with dsRNA. The different letters indicate significant differences (*p* < 0.05). Each point represents the mean ± SE from three independent experiments. (**B**) Survival rates of nymphs after injection with dsRNA. ** indicates significant differences (*p* < 0.01). Each point represents the mean ± SE from three independent experiments. (**C**) The mRNA expression levels of *NlECR*, *NlUSP*, and *NlHR3* at the 4th day after injection with dsRNA. The mRNA levels for each cDNA were normalized with respect to the 18s-rRNA mRNA level. Fold induction values of *CaMKII* genes were calculated with the ΔΔ*C*t equation and normalized to the mRNA level of *CaMKII* genes in the control (injected with dsGFP-200) which were defined as 1.0. dsGFP-200: nymphs injected with 200 ng dsRNA of *GFP*; dsNlCaMKII-200: nymphs injected with 200 ng dsRNA of *NlCaMKII*; and dsNlCaMKII-100: nymphs injected with 100 ng dsRNA of *NlCaMKII*.

In order to test the function of *CaMKII* in the development of *N. lugens*, dsRNA corresponding to the consistent region of eleven isoforms of *NlCaMKII* from *N. lugens* was synthesized and injected into third-instar nymphs with the concentration of 200 ng and 100 ng nymph^−1^. After injection, the transcript level of *NlCaMKII* at the 4th day in the treatment of dsCaMKII-200 and dsCaMKII-100 decreased by 67.2 and 27.6%, respectively (Figure 8A). The mortality depended on the dose of injected dsRNA. Compared to the mortality (about 18.0%) of the control injected with dsGFP-200, 43.3% of the injected nymphs died six days after injection with 200 ng/nymph dsRNA and the mortality increased to 77.7% at the 10th day. The injected nymphs presented normal phenotypes. No significant reduction in the survival rate after injection with 100 ng/nymph dsRNA was observed compared to the control group (Figure 8B). As reported in the lepidopteran insect *H. armigera*, *CaMKII* knockdown suppressed the 20E response gene expression, including the nuclear receptor EcRB1, the heterodimeric protein USP1, and transcription factors HR3 and BrZ7 involved in the synthesis of 20E. We conducted a RT-qPCR experiment to examine the expression levels of genes involved in the 20E in nymphs with silenced *NlCaMKII*. The expression of these genes did not decrease or increase when compared to the mRNA levels in the nymphs with injected dsGFP (Figure 8C).

## 3. Discussion

CaMKII, an evolutionarily conserved protein, is central to coordinate Ca^2+^ signaling and is responsive to numerous signal transduction pathways. It has a widespread localization and broad substrate specificity [7,22]. Species from fruit flies to humans encode alternative splice variants which are differentially targeted to phosphorylate the diverse downstream targets of Ca^2+^ signaling. Each isoform has a different affinity for a given substrate. The production of several versions of the same protein is a strategy that can be used by a cell to assign multiple roles to an enzyme [23]. All of the CaMKII isoforms have a catalytic domain; a regulatory domain, including CaM-binding and an autoinhibitory site; a variable segment; and a self-association domain [24]. Unlike CaMKI and CaMKIV, CaMKII has the ability to acquire Ca^2+^ independence, referred to as autonomy, when activated strongly by Ca^2+^ due to autophosphorylation. The acquisition of autonomy prolongs the active state of CaMKII and is likely to be critical for the generation of long-term potentiation, a strengthening of synaptic connections that underlies synaptic plasticity in learning and memory [25]. Previous research in insect CaMKII mainly concentrated on isolating the *CaMKII* gene or protein from the head or ventral nerve and studying its function in catalytic properties and central nervous system remodeling [11,14,18,20,26].

This article describes the molecular cloning of *CaMKII* from the whole body and the analyzing of its splice variants in three rice planthopper species. The obtained isoforms have a conserved core structure containing catalytic, regulatory, and association domains. Two main autophosphorylation sites (Thr-287 and Thr-306/Thr-307) and one additional site, Ser-315, are well conserved in planthoppers. The association domain contains highly conserved sequences as well as multiple variable inserts [22]. Isoforms differ primarily in their use of the variable inserted sequences. Among them, the isoform CaMKII-E, with no insert sequence, is the most abundant. Interestingly, we found that Insert 1 (20 aa) and Insert 2 (21 aa) in the variable regions of three planthopper species are almost identical and homologous to Inserts 1 and 2 in *Drosophila*, respectively. As reported in *P. americana*, the consensus phosphorylation site in Insert 1 is RSST [11]; while, in *Drosophila*, it was found to be RSTT [10]. The phylogenetic tree of deduced amino acid sequences showed that NlCaMKII isoforms are more closely related to that of LsCaMKII and slightly distinct from SfCaMKII.

Our findings showed that all CaMKII isoforms were expressed in the tested tissues and highly concentrated in the head. The RT-PCR analysis revealed that there was no major tissue specificity of isoform expression; however, the expression pattern and relative abundance of the isoforms varied among tissues. The NlCaMKII-A1 seems to be a minor form in *N. lugens*. In accordance with previous studies, *DmCaMKII* is expressed highly in fly heads and has a plethora of important functions in the nervous system [27,28]. Less is known about the functional difference among isoforms. In *Manduca sexta*, the *CaMKII* expression remained unaltered between the larval stages [29], whereas the CaMKII activity changed significantly during metamorphosis, a developmental regulation of CaMKII activity by alterations in calcium activation rather than by changes in the amount of *CaMKII* expression [11]. In *N. lugens* and *S. furcifera*, the amount of *CaMKII* expression altered slightly during development. Furthermore, we confirmed that the *NlCaMKII* expression did not change among different virulent populations. *NlCaMKII* was upregulated at the 4th day after TN1 population *of N. lugens* was infested on the IR56 rice. This result suggests that *NlCaMKII* may play important roles in the adaptation of *N. lugens* to resistant rice.

In the *NlCaMKII* RNAi experiment, we found that the maximum transcript reduction of 67.2% occurred at the fourth day with 200 ng/nymph of dsRNA and the mortality increased to 77.7 ± 0.5% by the 10th day. An injection of dsRNA with 100 ng/nymph only caused a 27.6% reduction, which was about half of that with 200 ng/nymph. A 67.2% suppression of *NlCaMKII* led to 77.7 ± 0.5% mortality at the 10th day, while 27.6% suppression did not influence nymph development. There were no significant differences in the average survival rates between the NlCaMKII-100 dsRNA pre-treated *N. lugens* (73.2 ± 3.7%) and the controls (81.9 ± 1.1%). This result was consistent with the report by Liu et al. that the RNAi efficiency for central nervous system genes was significantly lower than ubiquitously expressed genes [30], as RNAi is a method to downregulate gene expression at the transcript level, whereas the regulation of CaMKII mainly relies on activity-dependent genes at the protein level. One notable difference is that in *N. lugens*, the phenotype of a dead nymph was normal after an injection of dsNlCaMKII, whereas in *H. armigera*, 31.8% of larvae did not shed the old cuticle after the dsHaCaMKII injection. The behavioral changes that occur during the transformation from a crawling caterpillar into a flying moth require rebuilding of the central nervous system (CNS) [31,32] and most of the larval sensory neurons are replaced by new adult-specific ones during metamorphosis in *H. armigera*. As an essential signaling kinase involved in neuronal plasticity in insects, CaMKII may play a much more important role in holometabolous insect metamorphosis than in hemimetabolous insects. Specific primers and dsRNA could not be effectively designed for studying the function of isoforms individually by using RNAi. Further experiments, such as siRNA and CRISPR (Clustered regularly interspaced short palindromic repeats)/Cas-mediated knockout method, are needed to clarify the precise role of CaMKII isoforms in planthopper development and behavior.

## 4. Materials and Methods

### 4.1. Insects and Rice

Rice plants at the 3rd–5th leaf stages were used for this study. Experiments were performed using rice planthoppers—the brown planthopper (*Nilaparvata lugens* (Stål)), the white-backed planthopper (*Sogatella furcifera* (Horváth)), and the small brown planthopper (*Laodelphax striatellus* (Fallén)). The brown planthoppers were reared on rice variety Taichung Native 1 (TN1, a *N. lugens*-susceptible rice cultivar) in wire mesh cages at 28 ± 0.5 °C and 70 ± 5% relative humidity for a 16 h light/8 h darkness photoperiod. The white-backed planthopper and the small brown planthopper were collected in a field of China’s National Rice Research Institute, Fuyang, Zhejiang, China.

Five laboratory populations of *N. lugens* (TN1, ASD7, Mudgo, IR56, and IR42) with different virulences were used in this study and were reared on rice varieties Taichung Native1 (a *N. lugens*-susceptible rice cultivar), Mudgo (carrying the Bph1 gene), IR56 (carrying the Bph3 gene), and IR42 and ASD7 (carrying the bph2 gene), respectively, for more than 60 generations. The laboratory populations were named after the natal host rice lines that they were reared on. For the RNA extraction, thirty newly emerged individuals from each *N. lugens* population were randomly selected and 10 individuals were pooled into one group.

The tissues including salivary gland, gut, fat body, ovary, leg, wing, and cuticula were dissected under a Leica S8AP0 stereomicroscope from adults of *N. lugens*, *S. furcifera*, and *L. striatellus*, respectively. Each sample was repeated in biological triplicate. Hemolymph from *N.lugens* was collected as follows: *N. lugens* was attached dorsally onto a Petri dish using adhesive tape. The legs were removed, and the drops of hemolymph collected using a capillary glass. Tissue samples from 100 adults were pooled randomly into one group. All samples were collected in triplicate. The samples were frozen in liquid nitrogen and stored at −80 °C.

For the purpose of studying the CaMKII expression in the *N. lugens* response to resistant rice, newly emerged nymphs were collected from the TN1-*N. lugens* population and then randomly separated into two groups with 100 nymphs/group—one group was placed on rice variety Taichung Native1 and the other on IR56. Five samples were collected after 3 h, 6 h, 9 h, 12 h, 1 day, and up to 12 days of feeding, immediately frozen in liquid nitrogen, and stored at −80 °C for further use. All the experiments were performed with three independent biological replicates.

### 4.2. Total RNA Extraction and Reverse Transcription

Total RNA was isolated from *N. lugens*, *S. furcifera*, and *L. striatellus* using an RNase mini kit (Qiagen, Hilden, Germany), according to the protocol recommended by the manufacturer. The RNA was quantified, and the quality was verified by Nanodrop ND-1000 spectrophotometer (Nanodrop Technologies, Rockland, DE, USA). Complementary DNA (cDNA) was transcribed with a ReverTraAceqPCR RT Kit (Toyobo, Osaka, Japan) from five hundred nanograms of extracted total RNA, according to the manufacturer’s instructions, and stored at −20 °C. The 10× diluted cDNA (3.0 μL) was used as the template for the PCR.

### 4.3. Cloning of Full-Length cDNA Sequence and Sequence Analysis

Based on transcriptome database from the whole bodies of *N. lugens*, which was constructed in our laboratory, *S. furcifera*, and *L. striatellus*, which was constructed at Nanjing Agricultural University [33], the *CaMKII* gene was amplified respectively with a primer pair designed from the untranslated regions surrounding the 5′ and 3′ open reading frame (ORF) sequence using the NCBI primer design tool (www.ncbi.nlm.nih.gov/tools/primer-blast). All the primers used in this study are listed in Table 3 and were synthesized by Invitrogen Co., Ltd., Shanghai, China. RT-PCR was performed in a total volume of 25 μL containing 1 μL of RT products, 0.4 μM of each primer, 0.2 mM deoxyribonucleotide triphosphates (dNTP), using EX-Taq polymerase (TaKaRa, Shiga, Japan) for 40 cycles at 95 °C for 30 s, 58 °C for 40 s, 72 °C for 1 min, and final elongation of 72 °C for 7 min. Negative controls were carried out, in which reactions without the template cDNA were conducted. PCR products were analyzed by agarose gel (2.0%) electrophoresis. The PCR products from the agarose gel were purified using Wizard^®^ SV Gel and the PCR Clean-Up System kit (Promega Corp. Madison, WI, USA), cloned into the pCR2.1 TOPO vector (Invitrogen, China), and then the plasmid from positive colonies was sequenced with the M13 primer pair on an ABI Prism 3100 DNA sequencer (Bioshang, Shanghai, China).

Translations of cDNAs and predictions of the deduced proteins were conducted using DNAStar software (DNASTAR Inc., Madison, WI, USA). The sequences were aligned using ClustalW and then a phylogenetic tree was generated using the neighbor-joining method with MEGA 5.10 software http://www.megasoftware.net/. The robustness of the branches was assessed by performing a bootstrap analysis of 1000 replications. Exon placement and total gene size were determined by comparing *NlCaMKII* cDNAs to *N. lugens* genomic sequences (assembly GCA_000757685.1) [21]. The exon–intron structure was predicted by aligning the mRNA with the genomic DNA sequence using the Spidey program—http://www.ncbi.nlm.nih.gov/spidey/spideyweb.cgi.

### 4.4. Expression Analysis by RT-PCR and RT-qPCR

The *CaMKII* transcript patterns were analyzed in the tissues and three body parts—the head, thorax, and abdomen—of *N. lugens, S. furcifera*, and *L. striatellus*. The primer pair CaMKII-VR, which corresponded to the identical nucleotides flanking the variable region of *CaMKII*, were designed. RT-PCR was performed in a total volume of 25 μL containing 1 μL of RT products, 0.4 μM of each primer, 0.2 mM deoxyribonucleotide triphosphates (dNTP), using EX-Taq polymerase (TaKaRa, Shiga, Japan) for 40 cycles at 95 °C for 30 s, 58 °C for 50 s, 72 °C for 45 s, and final elongation of 72 °C for 7 min. PCR products were analyzed by agarose gel (2.0%) electrophoresis.

The *CaMKII* transcript levels were quantified in the tissues and development stages of *N. lugens, S. furcifera*, and *L. striatellus*, using an ABI 7500 Real-time PCR system (Applied Biosystems, Foster, CA, USA). RT-qPCR reactions were performed with a qPCR master mix SYBR^®^ Premix (Toyobo, Japan). The specific primer qCaMKII, which corresponded to identical nucleotides in the catalytic domain of *CaMKII*, was designed. A dissociation curve was performed at the end of each RT-qPCR run to ensure amplification specificity. The primer efficiency for RT-qPCR was checked with cDNA diluted 1:5 fold along 6 serial dilutions. The amplification program and melting curve analysis were used as previously described [34]. Each experiment consisted of three separate biological replicates, each of which comprised three technical replicates. The mRNA levels for each cDNA were normalized with respect to the 18s-rRNA mRNA level. Fold induction values of the target genes were calculated with the ΔΔ*C*t equation and normalized to the mRNA level of the target genes in the control which were defined as 1.0 [35].

### 4.5. RNAi Experiment and Bioassay

Template for dsRNA was amplified with the clone *NlCaMKII-A1*. T7 promoter sites (TAATACGACTCACTATAGGG) were added to the specific primers used for dsRNA synthesis. The PCR program used was 95 °C for 3 min, then followed by 40 cycles of 94 °C for 30 s, 60 °C for 45 s, and 72 °C for 1 min; finally, a 10 min extension step was performed. PCRs were conducted to yield dsDNA, followed by generation of dsRNA with the T7 MEGAscript RNAi kit (Ambion, Huntingdon, UK). The resultant dsRNA was purified and quantified using a Nanodrop2000 spectrophotometer (Thermo Scientific Inc., Waltham, MA, USA). The dsRNA was stored at −80 °C until use. The GFP gene (ACY56286) was used as a control.

The dsRNA microinjection experiment was conducted based on a previously described method [34]. The concentration of dsRNA was designated as a high dose (200 ng) and a medium dose (100 ng). In order to study the function of *NlCaMKII* in the development of *N. lugens*, third-instar nymphs were used for the microinjection. Thirty third-instar individuals were injected and reared on 30–35 day-old rice of Taichung Native1 in one cage. We performed the experiments in triplicate. Five individuals from one cage were randomly selected at 4 days after injection for RNA extraction and RT-qPCR. Three biological replicates were performed. GFP dsRNA was used for the injection in the control treatments. The effects of RNAi on the transcript expression of 20E response genes, including *NlECR*, *NlUSP*, and *NlHR3*, were quantified using RT-qPCR.

The survival rates and development defects of the 3rd instars after injection were observed at 24 h intervals for a duration of 10 days. All the results are presented as means ± standard errors. The differences between the *N. lugens* injected with different dsRNA and those injected with GFP dsRNA were assessed by Duncan’s multiple range test. Values of *p* < 0.05 were considered significant.

## Figures and Tables

**Table 1:** Characteristics of CaMKII isoforms from *N. lugens*, *L. striatellus*, and *S. furcifera.*

CaMKII Isoform	Accession Number	Full Length	ORF ^1^	CDS ^2^ Size	MW ^3^	Beginning AD ^4^	Insert	Clone No.
***N. lugens***								
NlCamMKII-A1	MH807664	2028	1719	572	64.3	SKYDVQG	4	1/52
NlCamMKII-B1	MH807665	1917	1608	535	59.4	S	3	2/52
NlCamMKII-C1	MH807666	1908	1599	533	59.9	SKYDVQG	1 + 2	3/52
NlCamMKII-C2	MH807667	1897	1587	529	59.4	KSG	1 + 2	1/52
NlCamMKII-C3	MH807668	1891	1581	527	59.2	S	1 + 2	2/52
NlCamMKII-D1	MH807669	1846	1536	512	57.5	SKYDVQG	1	1/52
NlCamMKII-D2	MH807670	1834	1524	508	57.1	KSG	1	2/52
NlCamMKII-D3	MH807671	1828	1518	506	56.9	S	1	3/52
NlCamMKII-E1	MH807672	1786	1476	492	55.5	SKYDVQG	NONE	14/52
NlCamMKII-E2	MH807673	1773	1464	488	54.9	KSG	NONE	8/52
NlCamMKII-E3	MH807674	1767	1458	486	54.8	S	NONE	15/52
***L. striatellus***								
LsCamMKII-A1	MH807675	1815	1668	556	62.6	S	4	1/51
LsCamMKII-B1	MH807676	1806	1659	553	61.3	SKYDVQG	3	1/51
LsCamMKII-B2	MH807677	1794	1647	549	60.8	KSG	3	1/51
LsCamMKII-B3	MH807678	1788	1641	547	60.6	S	3	1/51
LsCamMKII-C1	MH807679	1734	1587	529	59.4	KSG	1 + 2	4/51
LsCamMKII-C2	MH807680	1728	1581	527	59.2	S	1 + 2	4/51
LsCamMKII-C3	MH807681	1848	1701	567	62.8	S	1 + 3	1/51
LsCamMKII-D1	MH807682	1683	1536	512	57.6	SKYDVQG	1	2/51
LsCamMKII-D2	MH807683	1671	1524	508	57.1	KSG	1	2/51
LsCamMKII-D3	MH807684	1665	1518	506	56.9	S	1	3/51
LsCamMKII-E1	MH807685	1623	1476	492	55.5	SKYDVQG	NONE	4/51
LsCamMKII-E2	MH807686	1611	1464	488	59.7	KSG	NONE	10/51
LsCamMKII-E3	MH807687	1605	1458	486	54.8	S	NONE	17/51
***S. furcifera***								
SfCamMKII-A1	MH807688	1665	1545	515	58.1	SKYDVQG	4	2/46
SfCamMKII-B1	MH807689	1772	1650	550	60.8	S	3	3/46
SfCamMKII-C1	MH807690	1715	1599	533	59.9	SKYDVQG	1 + 2	2/46
SfCamMKII-C2	MH807691	1707	1587	529	59.3	KSG	1 + 2	2/46
SfCamMKII-C3	MH807692	1701	1581	527	29.1	S	1 + 2	3/46
SfCamMKII-D1	MH807693	1645	1524	508	57.1	KSG	1	3/46
SfCamMKII-E1	MH807694	1597	1476	492	55.5	SKYDVQG	NONE	10/46
SfCamMKII-E2	MH807695	1585	1464	488	54.9	KSG	NONE	9/46
SfCamMKII-E3	MH807696	1573	1458	486	54.8	S	NONE	14/46

^1^ ORF: open reading frame; ^2^ CDS: coding sequence; ^3^ MW: molecular weight; ^4^ Beginning AD: beginning amino acids of the association domain.

**Table 2:** Insert sequences in CaMKII from *N. lugens*, *L. striatellus,* and *S. furcifera*.

Insert name	Sequence ID: Sequence
***N. lugens***	
Insert 1	NlI1a: EDKSKGVVDRSSTVIAKDPE
Insert 1	NlI1b: EDKIKGGVDRSSTVIAKDPE
Insert 2	NlI2: ELRIVCPNKTISQISTNSYGS
Insert 3	NlI3: GIVGSCTPPQTPAAATTPTSKSAPVSFGAGAKPAATSALSLSAVLLQGAQ
Insert 4	NlI4:DVLSVPLRSASNGGTPSNGGIPSPSIRNRRLGTRLMCNQQDLLSVGLQQKRQDDSKYPEHLSNDSGEIGYQRLDCEPDNSE
***L. striatellus***	
Insert 1	LsI1:EDKSKGVVDRSSTVIAKDPE
Insert 2	LsI2:ELRIVCPNKTISQISTNSYGS
Insert 3	LsI3:GIVGSCTPPQTPAAATTNCGQPQTPTSKSAPVSFGFGSGSASVAKTSTTLSLSAVLLQGAQ
Insert 4	LsI4:VQQQKSGSNGIPSPSIRNRRLGTRLMSNQQELLTVQQKRHDDLKSPEHLSNDSGEIGYQRLDCGEQDNAE
***S. furcifera***	
Insert 1	SfI1:EDKVKGGVDRSSTVIAKDPE
Insert 2	SfI2:ELRIVCPNKTISQISTNSYGS
Insert 3	SfI3:GIVGNYTPPQTPAAATANCGQPQTPTSKSAPVSFGFGSGSTSSAAAAKTTTTLSLSAVLLQGAQ
Insert 4	SfI4:EHLSNDSGEISYQRLDCEQDNSE

**Table 3:** Primers used in this study.

Primer Name	Forward Sequence (5′–3′)	Reverse Sequence (5′–3′)	Amplicon Size (bp)
**Primers for CaMKII Clone**			
NlCaMKII	GGGTAGTTGCTGAGCGAAGAG	TACCAGAACGCACCGACAGA	1767–2028
LsCaMKII	TAGTTGTTGAGCGAGTGGATGG	ATCAACAGAGTGAGTGGGGAA	1605–1848
SfCaMKII	GTTGGTGAGCGAGTGGATGG	GTCTTGTCAGCGGGCAGTAG	1573–1772
**Primers for RT-qPCR**			
qCaMKII	AGTTATCGGTATGGCTGCTCC	TGGGGTGTTGAAGTTTCCGA	224
18s-RNA	CAAGTATCAATTGGAGGGCAAGT	GCACACAGTATACAGGCGTGA	342–368
NlUSP	CAGATGTGCGAGACCTGAAG	CAGCGCTGTGTACACCTTCT	58
NlHr3	AAGGAGACGTGACAGTGTGC	GGGAATTCGAGATGAGGTGT	121
NlECR	GCTGTGAAGCGAAAGGA	ATTCCACGCTGAAGTCG	126
**Primers for dsRNA Synthesis**			
dsCaMKII	T7-AGTGTCAACCACTGCCACAC	T7-AGGGTGGTACTACTATCTGTAGACT	668
dsGFP	T7-CCTGAAGTTCATCTGCACCAC	T7-TGATGCCGTTCTTCTGCTTGT	355
**Primers for** **RT-** **PCR**			
CaMKII-VR	GCC/AATTTTGACTACA/TATGCT	CTTTATAACTTCTTGCTTACGAG	153–414

## Supplementary Materials

Supplementary materials can be found at https://www.mdpi.com/1422-0067/20/12/3014/s1.

## Abbreviations

dsRNA	Double-strand RNA
CaMKII	Calcium/calmodulin-dependent protein kinase II
ORF	Open reading frame
GFP	Green fluorescent protein
RT-qPCR	Real-time quantitative polymerase chain reaction
RT-PCR	Reverse transcription polymerase chain reaction
RNAi	RNA interference
20E	20-Hydroxyecdysone
siRNA	Small interfering RNA
