# Supplementary material for: Molecular Characterization of Ca2+/Calmodulin-Dependent Protein Kinase II Isoforms in Three Rice Planthoppers—Nilaparvata lugens, Laodelphax striatellus, and Sogatella furcifera
Source: Int J Mol Sci. 2019 Jun 20;20(12):3014. doi: 10.3390/ijms20123014 (PMC6627886; doi:10.3390/ijms20123014)
Supplement: Supplementary file 1 [file ijms-20-03014-s001.zip › ijms-522390-SI/supplementaryfiles/Suppmentary agend.docx]

**Figure S1.** Phylogenetic tree of CaMKII isoforms in several insect species. Based on the protein sequence alignments, phylogenetic tree was constructed with Mega 5.0 software using maximum likelihood estimation method, together with 1000 bootstrap replicates and was rooted with Mus musculus sequence. The accession numbers of the insect CaMKII protein sequences are as: (See Table S1 for details of GeneDB accession numbers).

**Figure S2.** **(A)** RT-PCR products for developmental expression of *CaMKII* in the whole body of *N. lugens* and *S.furcifera*. **(B)** RT-qPCR analysis for developmental expression of *CaMKII*in the whole body of*N. lugens* and *S.furcifera*. The mRNA levels for each cDNA were normalized with respect to the 18s-rRNA mRNA level. Fold induction values of *CaMKII* genes in *N. lugens* and *S.furcifera* were calculated with the ΔΔCt equation and normalized to the mRNA level of *CaMKII* genes in 1^st^ instar nymphs of *N. lugens* and *S.furcifera* respectively which were defined as 1.0. Different letters above each bar indicate statistical difference by ANOVA followed by the Duncan’s Multiple Comparison test (P<0.05).cDNA templates were derived from 1^st^ nymph, and so on; F♀, newly emerged female adults; M♂, newly emerged male adults. Each point represents the mean ± SE from three independent experiments with ten individuals in each replicate.

**Figure S3.** **(A)** RT-PCR products for *NlCaMKII* gene from five *N.lugens* virulent populations. **(B)** RT-qPCR analysis for *NlCaMKII* gene of five *N.lugens* virulent populations. Each point represents the mean ± SE from three independent experiments. The mRNA levels for each cDNA were normalized with respect to the 18s-rRNA mRNA level. Fold induction values of CaMKII genes were calculated with the ΔΔCt equation and normalized to the mRNA level of CaMKII genes in TN1 population which were defined as 1.0.
