# Supplementary material for: Molecular Characterization of Ca2+/Calmodulin-Dependent Protein Kinase II Isoforms in Three Rice Planthoppers—Nilaparvata lugens, Laodelphax striatellus, and Sogatella furcifera
Source: Int J Mol Sci. 2019 Jun 20;20(12):3014. doi: 10.3390/ijms20123014 (PMC6627886; doi:10.3390/ijms20123014)
Supplement: Supplementary file 1 [file ijms-20-03014-s001.zip › ijms-522390-SI/supplementaryfiles/TABLE S1.docx]

|  | **Systematic Name** | **Accession No.** | **amino acids** |
| --- | --- | --- | --- |
| 1 | DmCaMKII-A | NP_726633.2 | 490 |
| 2 | DmCaMKII-B | NP_726634.1 | 509 |
| 3 | DmCaMKII-C | [NP_524635.3](https://www.ncbi.nlm.nih.gov/protein/NP_524635.3?report=genbank&log$=prottop&blast_rank=1&RID=7EK3FKU6015) | 490 |
| 4 | DmCaMKII-D | NP_726635.2 | 530 |
| 5 | DmCaMKII-E | NP_726636.2 | 509 |
| 6 | DmCaMKII-G | NP_001014696.1 | 516 |
| 7 | DmCaMKII-H | NP_001162831.1 | 490 |
| 8 | DmCaMKII-I | NP_001162832.1 | 530 |
| 9 | DmCaMKII-J | NP_001162833.1 | 531 |
| 10 | HaCaMKII | XP_021189043.1 | 510 |
| 11 | PaCaMKII-A | ADX05541.1 | 486 |
| 12 | PaCaMKII-B | ADX05542.1 | 507 |
| 13 | PaCaMKII-C | ADX05543.1 | 507 |
| 14 | PaCaMKII-D | ADX05544.1 | 528 |
| 15 | PaCaMKII-E | KC733178 | 526 |
| 16 | AmCaMKII | NP_001128422 | 524 |
| 17 | ApCaMKII | XP_001945479.2 | 601 |
| 18 | TcCaMKII | XP_966888.1 | 525 |
| 19 | BmCaMKII | NP_001177296.1 | 510 |
| 20 | MmCaMKII-A | AAH31745.1 | 478 |

Table S1. The details of accession numbers of the insect CaMKII protein sequences
