# Supplementary material for: Molecular Characterization of Ca2+/Calmodulin-Dependent Protein Kinase II Isoforms in Three Rice Planthoppers—Nilaparvata lugens, Laodelphax striatellus, and Sogatella furcifera
Source: Int J Mol Sci. 2019 Jun 20;20(12):3014. doi: 10.3390/ijms20123014 (PMC6627886; doi:10.3390/ijms20123014)
Supplement: Supplementary file 1 [file ijms-20-03014-s001.zip › ijms-522390-SI/supplementaryfiles/TABLE S2.docx]

Table S2. Three planthoppers variable domain protein sequences properties and similarities

| SeqID | NlI1b | LsI1 | SfI1 | NlI2 | LsI2 | SfI2 | NlI3 | LsI3 | SfI3 | NlI4 | LsI4 | SfI4 |
| --- | --- | --- | --- | --- | --- | --- | --- | --- | --- | --- | --- | --- |
| NlI1a | **90.0** | **100.0** | **90.0** | 10.0 | 10.0 | 10.0 | 20.0 | 20.0 | 25.0 | 20.0 | 20.0 | 15.0 |
| NlI1b |  | **90.0** | **95.0** | 15.0 | 15.0 | 15.0 | 20.0 | 20.0 | 25.0 | 25.0 | 20.0 | 10.0 |
| LsI1 |  |  | **90.0** | 10.0 | 10.0 | 10.0 | 20.0 | 20.0 | 25.0 | 20.0 | 20.0 | 15.0 |
| SfI1 |  |  |  | 10.0 | 10.0 | 10.0 | 15.0 | 25.0 | 25.0 | 25.0 | 20.0 | 10.0 |
| NlI2 |  |  |  |  | **100.0** | **100.0** | 19.0 | 19.0 | 19.0 | 23.8 | 19.0 | 9.5 |
| LsI2 |  |  |  |  |  | **100.0** | 19.0 | 19.0 | 19.0 | 23.8 | 19.0 | 9.5 |
| SfI2 |  |  |  |  |  |  | 19.0 | 19.0 | 19.0 | 23.8 | 19.0 | 9.5 |
| NlI3 |  |  |  |  |  |  |  | **72.0** | **70.0** | 14.0 | 12.0 | 13.0 |
| LsI3 |  |  |  |  |  |  |  |  | **85.2** | 13.1 | 13.1 | 17.4 |
| SfI3 |  |  |  |  |  |  |  |  |  | 12.5 | 14.1 | 13.0 |
| NlI4 |  |  |  |  |  |  |  |  |  |  | **65.7** | **91.3** |
| LsI4 |  |  |  |  |  |  |  |  |  |  |  | **78.3** |

CaMKII variable insert protein sequences from three planthoppers were aligned by clustalW analysis. Scores higher than 60 are in bold. Variable insert protein sequences listed in Table 2.
