# Supplementary figures and images for: Molecular Characterization of Ca2+/Calmodulin-Dependent Protein Kinase II Isoforms in Three Rice Planthoppers—Nilaparvata lugens, Laodelphax striatellus, and Sogatella furcifera
Source: Int J Mol Sci. 2019 Jun 20;20(12):3014. doi: 10.3390/ijms20123014 (PMC6627886; doi:10.3390/ijms20123014)

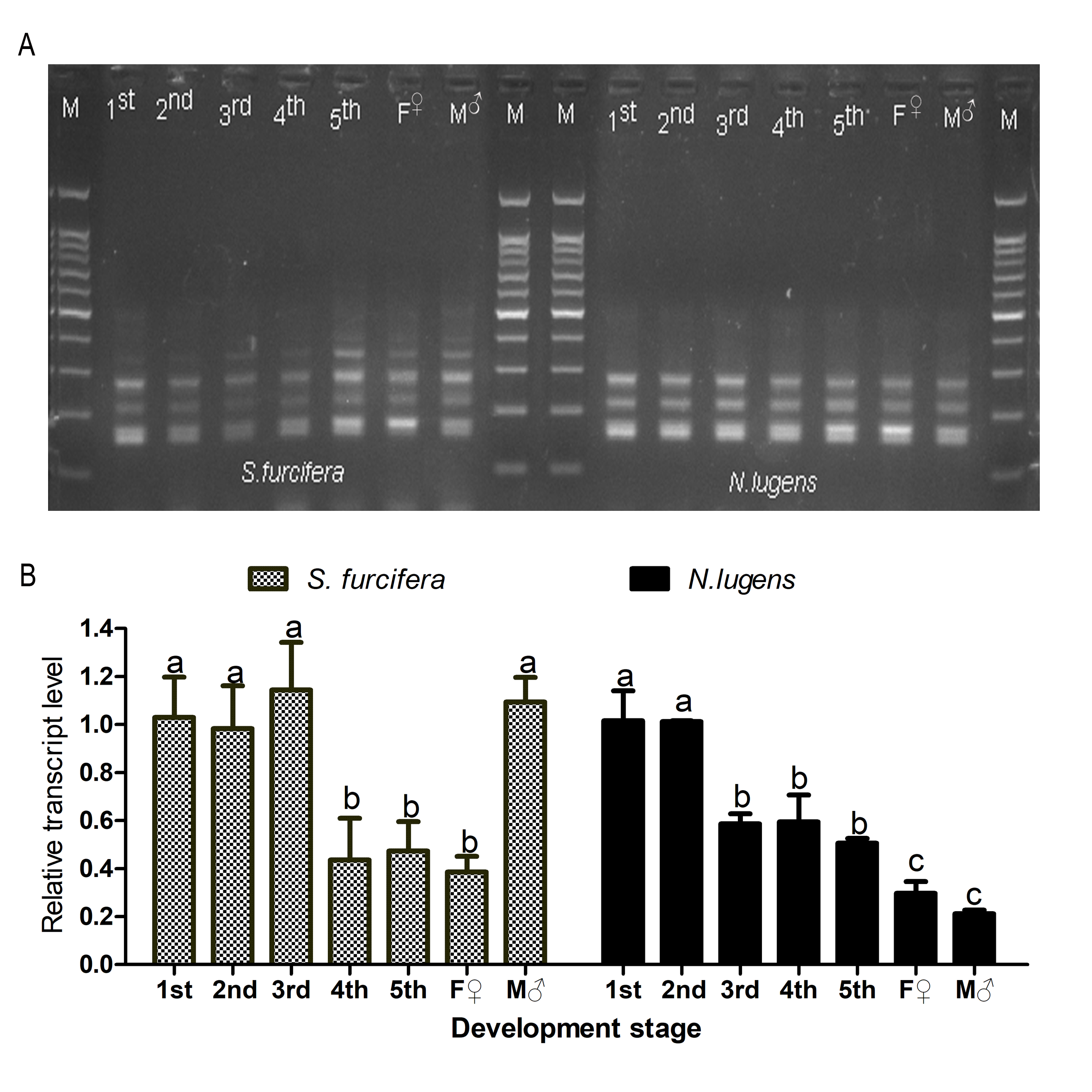

Supplement: Supplementary file 1 [file ijms-20-03014-s001.zip › ijms-522390-SI/supplementaryfiles/Fig S2.tif]

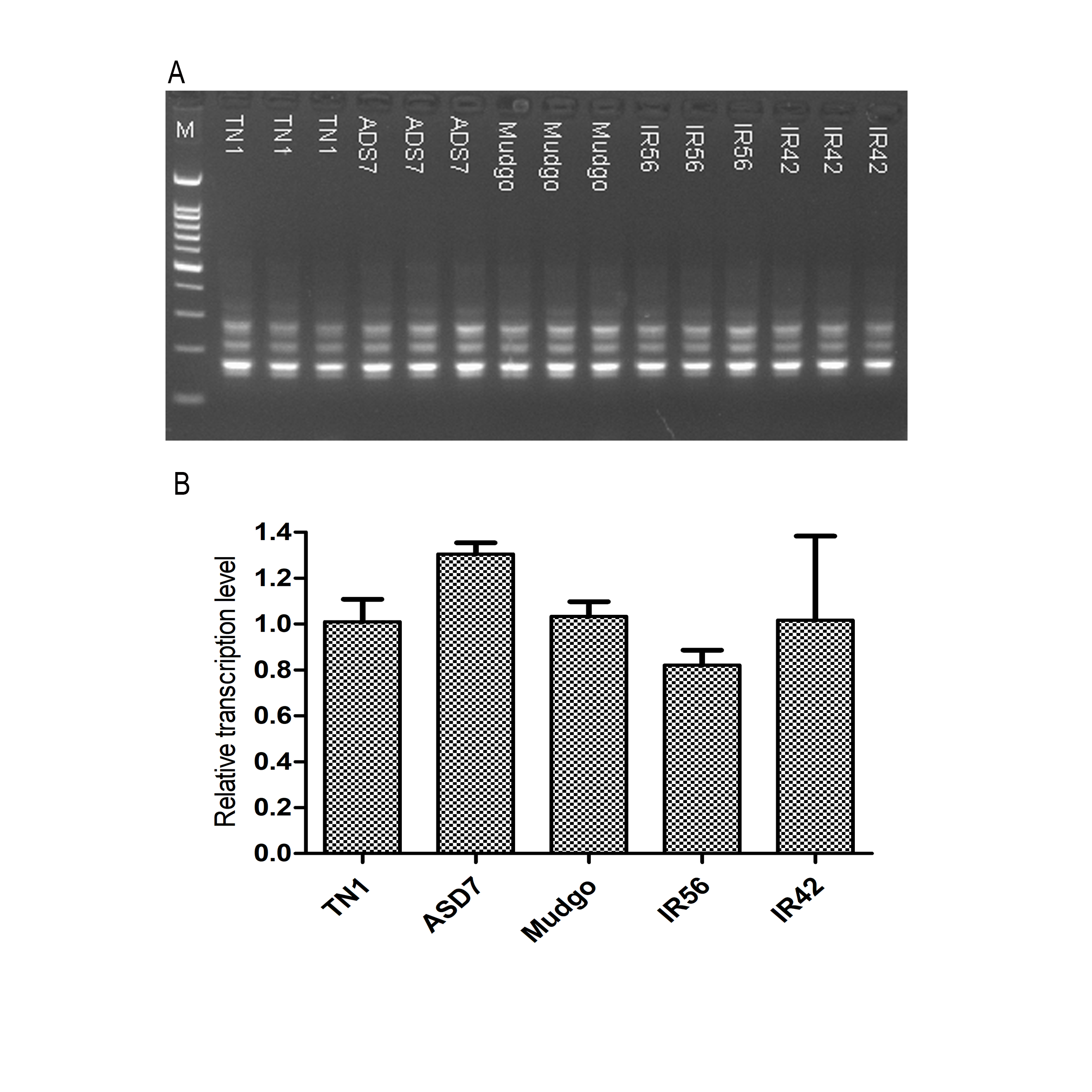

Supplement: Supplementary file 1 [file ijms-20-03014-s001.zip › ijms-522390-SI/supplementaryfiles/Fig S3.tif]
